# An *in vitro* screening assay for dental stain cleaning

**DOI:** 10.1186/s12903-016-0328-3

**Published:** 2017-01-09

**Authors:** Changxiang Wang, Robert Lucas, Anthony J. Smith, Paul R. Cooper

**Affiliations:** 1 Oral Biology, School of Dentistry, University of Birmingham, 5 Mill Pool Way, Edgbaston, Birmingham, B5 7EG UK; 2 GlaxoSmithKline Consumer Healthcare, St. George’s Avenue, Weybridge, Surrey KT13 ODE UK

**Keywords:** Enamel, Stain removal efficacy, Tooth whitening, Aesthetics, Toothpaste, Tooth colour, Surface roughness

## Abstract

**Background:**

The present study aimed to develop an *in vitro* model for stain removal from natural enamel for the assessment and comparison of oral hygiene products.

**Methods:**

Bovine teeth (*n* = 8 per group) were ground/polished to provide flat enamel specimens and ferric-tannate deposits were precipitated onto the enamel surfaces. The ferric-tannate stained enamel specimens were brushed using an *in vitro* tooth-brushing simulator with slurries containing commercially available toothpaste products, dental abrasive particles, and sodium tripolyphosphate (STP) solutions of different concentrations. The colour of the enamel surfaces was measured using a spectrophotometer before and after stain application as well as after the brushing treatments.

**Results:**

Differences in stain removal efficacy were found between the toothpastes categorised as whitening and non-whitening comprising of different types of dental abrasives (hydrated silica and alumina). A mean value of 27% for stain removal was detected for the three non-whitening toothpastes and 59% of stain removal was detected for the three whitening toothpastes after 1000 strokes. Compared with the slurry with Zeodent 113 abrasive alone, the addition of STP provided better performance for stain removal under the same brushing conditions (mean value of 62% for Zeodent 113 abrasive alone and 72% with the addition of 5% (w/w) STP after 1000 strokes). No difference was evident between the STP concentration of 5% (w/w) and 10% (w/w).

**Conclusions:**

The ferric-tannate/bovine enamel model reported here provides good stain retention, is rapidly and easily prepared, and is shown to be progressively and reproducibly sensitive to toothbrushing using different toothpastes and surfactant/chelating agent solutions. Importantly, it provides good discrimination between various oral hygiene products.

The stain removal assay reported here has considerable potential to enable comparative assessments of different toothpaste types in terms of their cleaning capabilities.

## Background

The natural colour of permanent teeth is largely determined by dentine and modified by the thickness and translucency of the overlying enamel [1]. The appearance of the teeth, particularly whiteness, is aesthetically important to individuals and tooth discolouration is a common dental patient complaint. Personal dissatisfaction with the appearance of the dentition has been reported to range from 17.9 to 52.6% [2–6] and the causes of tooth discolouration are multifactorial and are classified as extrinsic, intrinsic and internalised discolouration [7]. Intrinsic and internalised discolouration arise generally during tooth development or during disease and the more extensive localisation deep within the dental tissues constrains its reversal. Extrinsic staining of the tooth can arise from a variety of sources, such as smoking, red wine consumption, and the intake of cationic compounds, such as chlorhexidine or stannous salts [7–10]. Stain removal can be challenging as staining compounds can be bound to dental plaque and the acquired pellicle, as well as directly to the enamel surface [11]. Thus, oral hygiene strategies need to address both the removal of dental plaque and the extrinsic stain bound in these different ways.

While a variety of stain removal and tooth whitening procedures are used professionally in the clinic [12–15], they are relatively costly and labour intensive [16]. Furthermore, there is considerable demand for ‘over-the-counter’ (OTC) tooth-whitening products that can be integrated into a normal oral hygiene regime, and whitening toothpastes are commonly used for this [17]. In general, tooth-whitening toothpastes function by abrasive removal of extrinsic stain associated with the dental plaque, the acquired pellicle and the enamel surface together with the chemical cleaning action of constituents, such as sodium tripolyphosphate (STP), sodium pyrophosphate, sodium hexametaphosphate, hydrogen peroxide, as well as by the activity of the enzymes papain and bromelain [4].

Although randomised controlled trials (RCTs) can provide a high degree of evidence for cleaning efficacy, development of a robust laboratory model to evaluate the mechanisms of action and screen efficacy of whitening products or bleaching agents would be of significant benefit [18]. *In vitro* models allow rapid screening of a range of potential products; the most effective can subsequently be clinically assessed in RCTs. Various approaches have been used to investigate extrinsic stain removal by oral care products using a wide range of substrates such as polymethylmethacrylate, hydroxyapatite and enamel (human or bovine). Model stains, including tea, coffee, gastric mucin, soy broth, bacteria, chlorhexidine, saliva, tea with orange II Rhodamine, blood, cola, wine, tobacco and ferric-tannate, have all been used [19–24]. One of the most commonly applied *in vitro* approaches uses cut, polished and acid etched bovine enamel specimens stained with a solution containing a combination of coffee/tea/gastric mucin/Sarcina lutea turtox [19]. The stained specimens are then repeatedly brushed for a period of time using an automated approach with toothpaste slurries. Furthermore, a recent model has utilised a ferric-tannate coating deposited onto highly polished sintered hydroxyapatite discs and was reported to mimic the daily control of pellicle growth, maturation and staining [24].

A significant limitation of current protocols is the weak bonding between the stain and substrate constraining discrimination between different cleaning products and regimes. A further issue is the need for a staining time of several days necessary to achieve sufficient stain build-up for high-throughput analysis and discrimination of cleaning regimes. There is also a need to better model stain interactions with natural enamel to provide good clinical relevance. Subsequently, the aim of this study was to establish a relatively rapid and reproducible staining protocol with appropriate bonding to enamel, which would allow high-throughput assessment of the cleaning ability, and discrimination between oral care products, following brushing.

## Methods

### Specimen preparation

Previously, a model has been reported to simulate up-to-24 h pellicle formation generated by precipitating an iron (III) complex with tannic acid from aqueous solution directly onto polished hydroxyapatite discs [24]. In the present study, this model was modified to investigate the use of natural enamel specimens and to study the influence of specimen surface finish, which can influence stain retention.

Bovine teeth were collected and stored in 0.1% (w/w) thymol (Sigma-Aldrich, UK) solution at 4 °C prior to use. Bovine enamel specimens (approximate 18 mm × 12 mm) were prepared from tooth crowns by dissection using a diamond-edged saw and embedded in blocks of epoxy resin (Ø25 mm) (Buehler, UK). To investigate the effects of surface roughness on the retention and removal of the stain, eight bovine enamel specimens per treatment group were prepared to either: **a)** 600-grit SiC and 3 μm diamond finish (Polished surface group), **b)** 400-grit SiC ground finish (Partially roughened surface group), or **c)** 280-grit SiC ground finish (Roughened surface group) with 5 min ultrasonication in water following each treatment for removal of any residual grinding/polishing materials. A Phoenix Beta Grinder/Polisher (Buehler, UK) was used with SiC abrasive discs (Buehler, UK) for sample preparation. Samples were then surface profiled using a Talysurf Series 2 inductive gauge profilometer (Taylor-Hobson, UK), which has a 1 mm range in the z-axis and a resolution of 16 nm. The inductive gauge profilometer uses a conical probe with 2 μm diamond tip to accurately measure surfaces at the sub-micron level. Linear line profiles (2D) were measured on the surfaces at a measurement speed of 0.5 mm/s and with points spacing of 0.25 μm, and the arithmetic mean surface roughness (Ra) values were calculated (μltra version 5.1.14, Taylor-Hobson, UK) prior to staining.

### Tooth stain development

Freshly combined solutions (0.1% (w/w) of diammonium iron (II) sulphate 6-hydrate (Sigma-Aldrich) and 0.1% (w/w) tannic acid (ACS reagent, Sigma-Aldrich) are initially colourless, but form a dark colloidal iron (III) tannic acid complex (“ferric-tannate”) on contact with air, which resembles a dietary tannin staining. The fresh mixture was applied as successive layers on the enamel specimens, with each layer being dried at 40 °C in an oven (D-63450 Hanau, Heraeus Instrument (now Kendro Laboratory Products Ltd), Germany) for 10 mins before application of the subsequent layer. For the first layer, a 40 μl aliquot of the solution was pipetted onto each specimen and allowed to spread evenly over the specimen surface before drying. For the subsequent layers (up to a maximum of 9), 10 μl aliquots of the solutions were applied before drying. A total of 1, 3, 5, 7 and 10 cycles of successive stain deposition layering were investigated to study *in vitro* stain removal efficacy. To mimic the presence of an acquired pellicle layer, one of four different acidic polymers of Carbopol (Lubrizol Advanced Materials, Inc., USA), Carrageenan (Cargill, Incorporated, USA), Gantrez (Ashland, USA) and Xanthan (Cargill, Incorporated, USA) was incorporated and applied to assess stain accumulation and removal. Polymers were incorporated by either: **a)** applying a 40 μl aliquot of 0.1% (w/w) solution of the polymer followed by application of the ferric-tannate stain, or **b)** by reducing the amount of tannic acid in the stain by 50% and replacing it with an equivalent amount of one of the four acidic polymers as described above for the application of the stain.

### Stain removal

The stained enamel specimens were mounted in two brushing channels of an *in vitro* brushing simulator, as previously reported [25]. Oral B P35 medium toothbrushes were used for the brushing. Specimens were double brushed at a brushing speed of 120 rpm and a temperature of 20 °C was maintained throughout the entire brushing procedure. 150 g toothpaste/abrasive slurry was used in each channel and a brushing load of 150 g was applied. Specimens were double brushed sequentially for up to 5000 strokes. After brushing for the requisite number of strokes, specimens were thoroughly rinsed with water prior to colour evaluation (as described below). The abrasive slurries examined in the present study included **a)** a range of toothpaste slurries (see Table 1 for a list of products used) prepared with 25 g toothpaste in 40 ml water, **b)** 15% (w/w) of Zeodent 113 (Huber Corporation, USA) silica abrasive in 0.5% (w/w) Hercules 7 MF Carboxymethyl Cellulose (CMC) (Hercules Incorporated, USA) containing 10% (w/w) Glycerol (VWR International BVBA, Belgium), with or without the addition of 5% (w/w) and 10% (w/w) sodium tripolyphosphate (STP) (Sigma-Aldrich), **c)** 10% (w/w) aqueous STP, and **d)** water as a control. Zeodent 113 is a precipitated silica, which has commonly been employed as a toothpaste abrasive [25], with a 15 μm mean particle size (Mastersizer 2000, Malvern Instruments Ltd, United Kingdom).

**Table 1:** Toothpastes studied

Group	Toothpaste	Type/relevant ingredients	Manufacturer
A	Aquafresh Multi-Action Whitening	Whitening/hydrated silica, pentasodium triphosphate	GlaxoSmithKline Consumer Healthcare, Brentford, UK
B	Arm and Hammer Advanced Whitening	Whitening/hydrated silica, sodium bicarbonate	Church & Dwight UK Ltd., Kent, UK
C	Colgate Cavity Protection	Non-whitening/dicalcium phosphate dihydrate, tetrasodium pyrophosphate	Colgate-Palmolive, Guildford, UK
D	Colgate MaxWhite	Whitening/hydrated silica, white micro crystals, tetrasodium pyrophosphate	Colgate-Palmolive, Guildford, UK
E	Crest Cavity Protection	Non-whitening/hydrated silica, trisodium phosphate	Procter & Gamble UK, Weybridge, UK
F	Crest Whitening Expressions	Whitening/hydrated silica	Procter & Gamble, Cincinnati, USA
G	Pearl Drops Daily Whitening Toothpolish	Whitening/hydrated silica, alumina, tetrapotassium pyrophosphate	Church & Dwight UK Ltd., Kent, UK
H	Signal White Now	Whitening/hydrated silica, trisodium phosphate	Unilever Deutschland, Hamburg, Germany

The cleaning ability of eight dentifrices to remove stain pellicle was determined in parallel by a laboratory testing method (PCR) commonly used within the oral hygiene industry [19]. The results were compared with the stain removal of the same dentifrices using the current stain removal methodology.

Images of the enamel surfaces before staining and post-stain removal with 5000 brush strokes were also captured by using a camera (Nikon D7000, Nikon Corporation) to demonstrate stain removal effects.

### Colour evaluation

The colour of each tooth specimen (L*, a*, b*) was measured using a calibrated spectrophotometer (Minolta CM-2600d) before staining (=initial), after up to 10 cycles of stain application and after the brushing treatments. All surfaces were consistently dried by carefully wiping with a soft tissue prior to colour measurements. The L* value represents the value of ‘brightness/darkness’ of a colour, such that a perfect black body has an L* value of zero and the perfect reflecting diffuser has an L* values of 100. The a* and b* represent two colour axes, with a* the red-green axis and b* the yellow-blue axis [25].

Removal of the stain was assessed using the following equation:

$$ \% Removal=\frac{L*(Brushed)-L*(Stained)}{L*(Initial)-L*(Stained)}\times 100 $$

Where L* (Initial) is the brightness before staining; L* (Stained) is the brightness after 10 cycles of stain application and L* (Brushed) is the brightness after toothbrushing for the requisite number of strokes with water or toothpaste/abrasive slurry.

### Pellicle cleaning ratio (PCR)

The pellicle cleaning ratio (PCR) for the same batch of toothpastes was undertaken and the details of the methodology have been previously reported elsewhere by Stookey et al. [19]. The PCR data were compared with those from the present methodology.

### Statistical analyses of the data

Data were analysed by single factor ANOVA with a significance level of *p* ≤ 0.05 applied. Pearson correlation coefficient was used to determine the linear relationship between two variables, and is denoted by r. Given a value between +1 and −1 inclusive, where one is perfect positive correlation, 0 is no correlation, and −1 is perfect negative correlation.

## Results

### Effects of enamel surface finish on stain removal

**Fig. 1:**
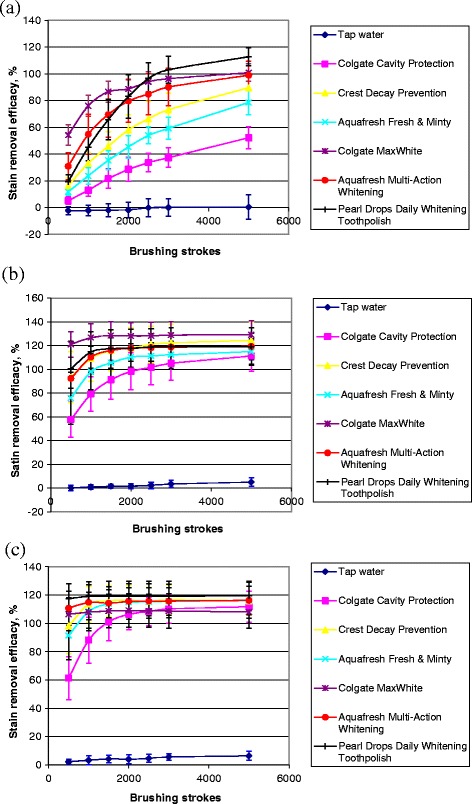
Effects of initial surface roughness of bovine enamel specimen surfaces on the stain removal efficacy after brushing with the tested toothpaste slurries and water for up to 5000 brush strokes, *n* = 8, mean ± standard deviation. **a** Roughened enamel surface group; **b** Partially roughened enamel surface group; **c** Polished enamel surface group

Enamel specimens of mean surface roughness values of approximately 0.4 μm (280-grit ground), 0.15 μm (400-grit ground) and 0.02 μm (diamond polished) were compared with respect to their susceptibility to stain removal after brushing with either water or commercial toothpastes (Fig. 1). Brushing with water resulted in minimal stain removal even after 5000 brush strokes with values of less than 1%, 5% and 10% for the 280-grit ground, 400-grit ground and diamond polished enamel surfaces, respectively. Stain removal was considerably enhanced after brushing with the commercial toothpastes with an inverse relationship between stain removal and specimen surface roughness. Statistically significant differences (*p* < 0.05) were found in the *in vitro* stain removal efficacy for the majority of data between 280-grit ground and 400-grit ground, and 280-grit ground and polished enamel specimens. However no significant differences (*p* > 0.05) were found in the *in vitro* stain removal efficacy between 400-grit ground and polished enamel specimens except for brushing with Colgate MaxWhite toothpaste. The numbers of brush strokes required to remove virtually all of the stain were 3000 or more for the 280-grit ground enamel specimens, 1000 or fewer for the 400-grit ground enamel specimens and 500 strokes or fewer for the diamond polished enamel specimens, respectively. There was a trend for greater stain removal with the whitening toothpastes using the same number of brush strokes. Due to the superior stain retention and greater ability to discriminate between individual commercial toothpastes, the 280-grit ground finish was used for all subsequent studies.

**Fig. 2:**
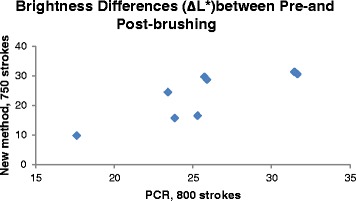
Comparisons between the PCR [19] and the present methodologies for the brightness changes between pre- and post-brushing after brushing with the toothpastes slurries, *n* = 8, mean value

The brightness differences for the 280-grit ground bovine enamel surfaces between the pre- and post-brushing are presented in Fig. 2. Due to the number of brush strokes (800 for PCR methodology [19], 500 and 1000 strokes for present methodology), an average difference for the 500 and 1000 brush stroke procedures (denoted as 750 strokes in Fig. 2) was used for the comparisons between the two methods. No statistically significant differences were detected between the two test methods when brushed with Arm & Hammer Advanced Whitening, Crest Whitening Expressions, Colgate MaxWhite, Pearl Drops Daily Whitening Toothpolish, and Signal White Now toothpastes. Statistically significant differences were however identified when brushed with Crest Cavity, Colgate Cavity, and Aquafresh Multi-Action Whitening, with more stain being removed by using the PCR method, indicating that better retention of stain was achieved using the present methodology. Pearson correlation coefficient result (*r* = 0.82) showed a strong positive linear relationship between these two methods.

**Fig. 3:**
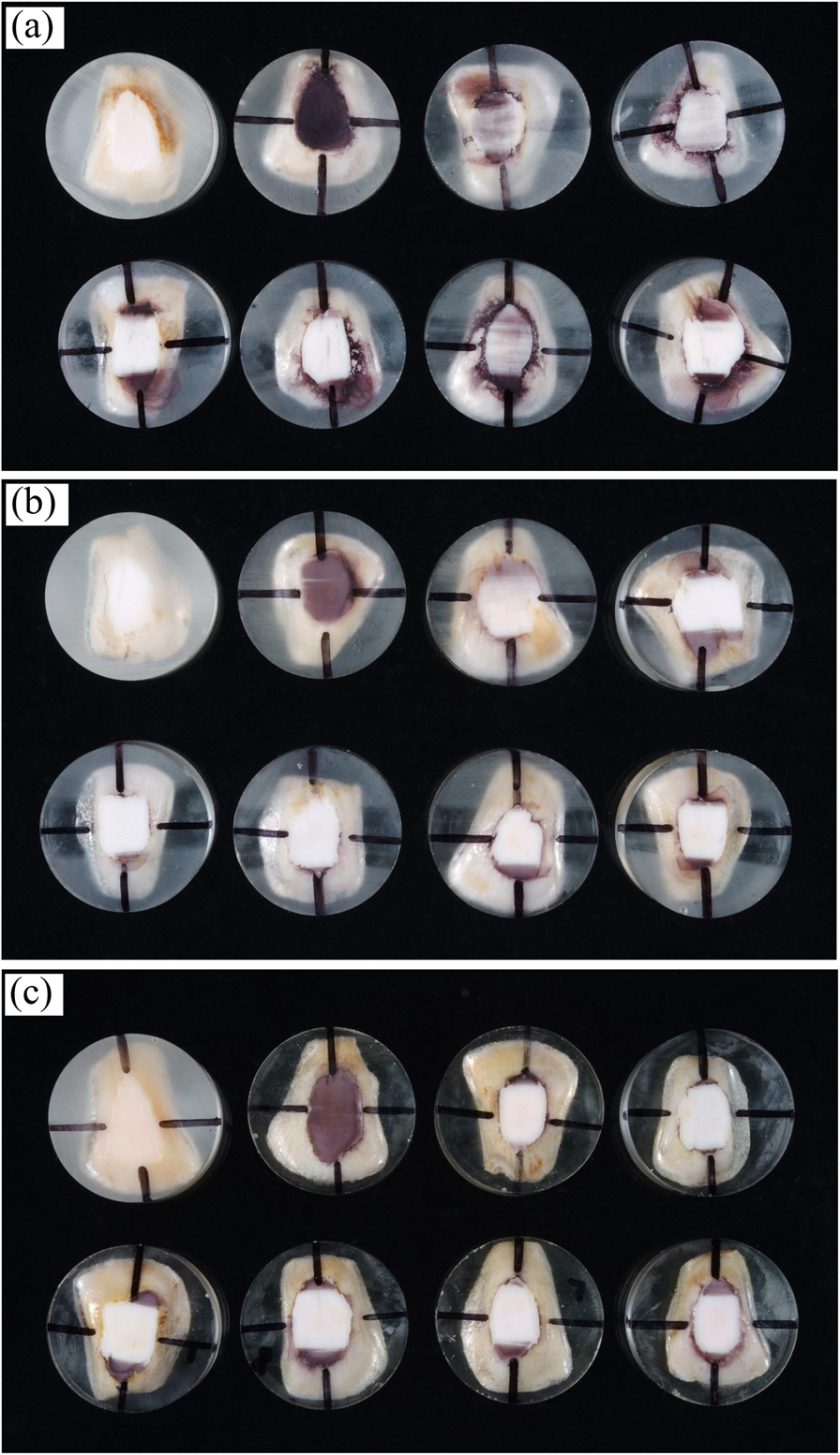
Images of enamel surfaces before staining, after staining and post-stain removal with 5000 brush strokes. **a** Roughened enamel surface group; **b** Partially roughened enamel surface group; **c** Polished enamel surface group. *Upper row* (left to right): before stain; stain brushing with water; stain brushing with Colgate cavity Protection toothpaste; stain brushing with Crest Decay Prevention toothpaste; *Lower row* (left to right): stain brushing with Colgate MaxWhite toothpaste; stain brushing with Aquafresh Multi-Action Whitening toothpaste; stain brushing with Aquafresh Fresh & Minty toothpaste; stain brushing with Pearl Drops Daily Whitening Toothpolish toothpaste

Figure 3 shows the images of enamel surfaces before staining and post-stain removal with 5000 brush strokes. Upon inspection it is apparent that relatively little stain remained on the roughened enamel surface group after brushing with the three non-whitening toothpastes, and no stain remained after brushing with the three whitening toothpastes. Relatively little stain remained on the partially roughened enamel surface group only after brushing with the Colgate Cavity Protection toothpaste, but no stain was apparent after brushing with the other tested toothpastes. No stain was apparent on the polished enamel surface group after brushing with all the tested toothpastes.

### Effects of successive layers of stain deposition

**Fig. 4:**
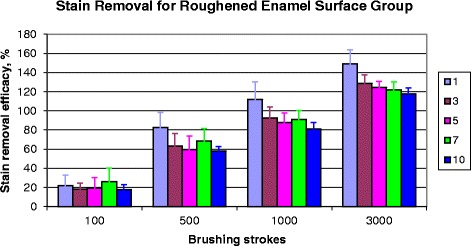
Stain removal efficacy for 280-grit ground enamel specimens with varying numbers of stain layers after brushing with 15% w/w Zeodent 113 precipitated silica abrasive slurry for up to 3000 brush strokes, *n* = 8, mean ± standard deviation

There was a linear decrease in surface brightness of specimens with deposition of up to 10 successive layers of stain (*r* = 0.98). The effects of the number of layers of stain deposition on stain removal after brushing were assessed by brushing with a typical toothpaste precipitated silica abrasive slurry (15% w/w Zeodent 113) (Fig. 4). Significant differences (*p* < 0.05) in stain removal efficacy were detected between the first and successive cycles of stain deposition after more than 100 brushing strokes, no significant differences (*p* > 0.05) were detected between the staining cycles of 3, 5, 7 and 10. However, inter-sample variation tended to decrease with increasing number of layers of stain deposition.

### Modelling the presence of acquired pellicle by application of acidic polymers

**Fig. 5:**
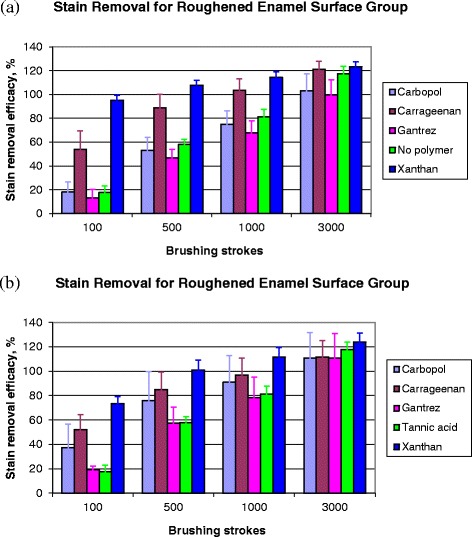
Modelling the application of an acquired pellicle prior to stain deposition on 280-grit ground finish enamel specimens using a variety of acidic polymers, *n* = 8, mean ± standard deviation. **a** by application of one layer of acidic polymers followed with 10 layers of tannate stain; **b** by replacing half of the tannic acid stain component with acidic polymers for the precipitation of 10 layers of stain

The application of an acquired pellicle prior to stain deposition (10 layers) was modelled on 280-grit ground finish enamel specimens using a variety of acidic polymers. Their effects on stain removal efficacy were subsequently assessed after brushing with a precipitated silica (typically present in commercial toothpastes) abrasive slurry (15% (w/w) Zeodent 113) (Fig. 5a). Significant differences (*p* < 0.05) in stain removal efficacy were observed between the polymers applied with a ranking order of stain retention of Carbopol and Gantrez as the best, then no polymer incorporation followed by Carrageenan and Xanthan being the poorest for stain retention. Treatment with the polymers Gantrez and Carbopol prior to stain deposition resulted in small decreases in stain removal efficacy after brushing, while Carrageenan and Xanthan negatively impacted on stain retention with greater stain removal observed. Application of the polymer by replacing 50% of the Tannic acid with the four acidic polymers during stain deposition generally decreased stain retention following brushing compared with the control (Fig. 5b). There were significant differences in stain removal efficacy between the presence of the four polymers versus no polymer inclusion (100% tannic acid) in the stain (*p* < 0.05). Following brushing with 15% (w/w) Zeodent 113, the rank order of stain retention was Gantrez and no polymer as the best, then Carbopol and Carrageenan and Xanthan representing the worst. Only Gantrez increased stain retention after brushing for 100, 500 or 1000 strokes, although such differences were not apparent at 3000 brush strokes. The application of the other polymers provided lower or equal levels of stain retention compared with the control after the various numbers of brush strokes.

### STP effect on stain removal

**Fig. 6:**
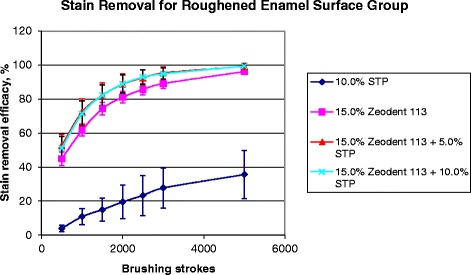
Stain removal efficacy for stained bovine enamel specimens after brushing for up to 5000 brush strokes with 10.0% (w/w) STP alone, 15.0% (w/w) Zeodent 113 alone, and 15.0% (w/w) Zeodent 113 with 5.0% (w/w) STP and 10.0% (w/w) STP, *n* = 8, mean ± standard deviation

STP is commonly used in oral hygiene products to facilitate stain removal [2]. Brushing of stained enamel specimens with STP both in the absence and presence of silica abrasive (15% (w/w) Zeodent 113) enhanced stain removal (Fig. 6). Brushing with 10% (w/w) STP alone removed approximately 35% of the stain after 5000 strokes and addition of Zeodent 113 abrasive further increased stain removal.

Statistically significant differences in stain removal efficacy were detected between the stained samples brushed with water (Fig. 1a) and 10% (w/w) STP. There were significant differences (*p* < 0.05) in stain removal when the stained enamel specimens were brushed with the three slurries comprising 15% (w/w) Zeodent 113 abrasive particles (+/− STP), while no significant differences were observed between the slurries comprising either 15% (w/w) Zeodent 113 abrasive plus 5% (w/w) or 10% (w/w) STP (*p* > 0.05). The three slurries with 15% (w/w) Zeodent 113 abrasive particles (+/− STP) exhibited the most effective stain removal as measured by brush strokes and linear responses for stain removal were observed during the first 500–1500 brush strokes (*r* > 0.98). The 10% (w/w) STP solution alone (without abrasive particles) showed a reasonably linear response for stain removal throughout the entire range of 500–5000 brush strokes (*r* = 0.97).

## Discussion

The *in vitro* stain methodology developed in this study provides a relatively inexpensive, easily performed, rapid and reproducible staining protocol which allows examination of stain bound to a natural enamel substrate. Of the many laboratory methods used to evaluate the cleaning potential of toothpaste and toothpaste ingredients, that developed by Stookey et al. [19] (PCR methodology) is commonly used by the oral care industry. However it requires a time consuming stain deposition protocol with opportunities for reproducibility issues when used at different test sites. The data presented here demonstrate good agreement between our stain methodology and the PCR methodology in terms of brightness changes after brushing using the same batches of toothpastes. The current study has also demonstrated the retention of the stain after toothbrushing in the presence of either standard toothpastes or whitening toothpastes as well as the most common chemically active compound in whitening toothpastes, STP. Furthermore it demonstrates the ability of the methodology to discriminate between these various oral hygiene products.

Model stains have been previously described [19–23] for simulation of the extrinsic staining of the teeth. However, one of the main disadvantages of these staining approaches has been that the stain properties could not be directly compared due to the differences in materials used, i.e., different brand/supplier of reagents used in different studies. However this ferric-tannate stain now provides a reliable standardised model for stain removal purposes in which the stain properties can be controlled due to the chemically defined nature of the stain deposits.

In the previously reported PCR methodology [19], acid etching of polished enamel surfaces has been used to expedite stain accumulation and adherence, although the reproducibility of the etching process can be difficult to accurately reproduce. In the present study, stain could be most readily removed from the diamond polished bovine enamel surfaces, followed by the 400-grit ground enamel bovine surfaces while the stain on the 280-grit ground bovine enamel surfaces was retained to the greatest extent. The surface roughness of enamel specimens can be reproducibly achieved with grinding/polishing protocols contributing to the reproducibility of the presently reported methodology. The surface roughness of the tooth is also of clinical importance, particularly in respect to bacterial retention. A threshold surface roughness for bacterial retention of 0.2 μm has been reported [26]. Indeed, the mean surface roughness values for 400-grit ground bovine enamel were approximately 0.15 μm, which is below the threshold roughness of 0.2 μm and the stain was more readily removed from the 400-grit ground surfaces than from the 280-grit ground bovine enamel surfaces, the latter of which had a mean surface roughness of approximately 0.4 μm. The polished bovine enamel surfaces had mean surface roughness values of approximately 0.02 μm, which is ten-fold less than the threshold roughness for bacterial retention and the stain on these polished surfaces was removed much more readily than that from the 400-grit ground and 280-grit ground bovine enamel surfaces.

The acquired enamel pellicle (AEP) which occurs in vivo results from rapid binding of salivary constituents after saliva contacts a newly exposed enamel surface and reportedly contributes to enamel protection [11]. It is generally understood that the AEP reduces friction between teeth and between teeth and the oral mucosa. It has also been reported that the pellicle enables the initial attachment of bacteria to the tooth surface, which is the first step in plaque formation [11]. In the present study, four acidic polymers were used to mimic an AEP layer on the 280-grit ground bovine enamel surfaces, by either direct application of a thin layer of the acidic polymer prior to application of the ferric-tannate stain or by partly replacing tannic acid with acidic polymers for the stain formation. Appreciable differences in stain removal efficacy were found between the four acidic polymers with a rank order of stain retention of Gantrez > Carbopol > Carrageenan > Xanthan. Gantrez on both occasions showed a very similar pattern of stain removal, indicating its potential to mimic an AEP layer prior to the stain deposits.

STP, a sodium salt of triphosphoric acid with surfactant and chelating properties, has been used in whitening toothpastes for stain removal. *In vitro* studies with crystalline hydroxyapatite (HA) powder showed that STP was effective in removing existing stain and impeding stain formation through inhibition of the adsorption of salivary proteins or tea stain and the desorption of existing protein and stain from HA surfaces [16, 27]. In vivo trials have also reported significant extrinsic stain removal efficacy for a STP containing dentifrice versus the control [28] as well as a reduction in staining by a chewing gum which contained STP [29]. In the present study, the effects of STP on *in vitro* stain removal efficacy were examined both in combination with abrasive particles and alone using 280-grit ground finish bovine surfaces. Approximately 35% of the stain was removed when brushing with 10.0% (w/w) STP alone after 5000 strokes, while a control of water resulted in minimal stain removal (less than 1%). The addition of 5.0 (w/w)% or 10.0 (w/w)% STP to a slurry of 15.0 (w/w)% Zeodent 113 abrasive improved stain removal compared with a 15.0 w/w% Zeodent 113 abrasive slurry alone for the same number of brush strokes. These data also highlight the efficacy of STP in contributing to stain removal during oral hygiene measures. However, no differences were observed between the STP concentration of 5.0 (w/w)% and 10.0 (w/w)%. Interestingly, a lack of influence of STP concentration on stain desorption from HA has also been reported elsewhere [27]. Compared with the 10.0 (w/w)% STP solution alone (without abrasive particles), the slurries with 15.0 w/w% Zeodent 113 abrasive particles (+/− STP) showed significantly higher stain removal efficacy, demonstrating that the abrasive was mainly responsible for the removal of extrinsic stain. Similar results have been reported by others when testing different types of whitening toothpaste products [30].

The present ferric-tannate/bovine enamel (280-grit ground finish) methodology provides an easily performed and reproducible model, which can be readily established in laboratories. The chemically defined nature of the stain contributes to reproducibility of the methodology as does the enamel sample preparation protocol. The rapid stain deposition (about 2 h for 10 cycles) facilitates the use of the methodology for rapid screening of large numbers of oral care products to target those products most suitable for testing under clinical conditions. Importantly, it allows good discrimination between various oral care products, including those used for tooth whitening purposes.

With the growth in the desire for whiter teeth by consumers and patients, toothpaste manufacturers aim to maximise the cleanability of enamel extrinsic staining whilst minimising possible damage (wear) to dental hard tissues. Therefore, it is important to investigate the wear and roughness in parallel to stain removal when using the present methodology.

## Conclusion

We conclude that this *in vitro* ferric-tannate stain removal assay provides an easily performed and reproducible screening assay for a variety of oral care products, which can be established in most laboratory settings. Acidic polymers can be applied for the AEP simulation prior to stain formulations.

## Abbreviations

AEP: Acquired enamel pellicle
HA: Hydroxyapatite
OTC: Over-the-counter
PCR: Pellicle cleaning ratio
RCTs: Randomised controlled trials
STP: Sodium tripolyphosphate

## References

[1] Sharif N, MacDonald E, Hughes J, Newcombe RG, Addy M (2000). The chemical stain removal properties of ‘whitening’ toothpaste products: studies *in vitro*. Br Dent J.

[2] Joiner A (2010). Whitening toothpastes: a review of the literature. J Dent.

[3] Alkhatib MN, Holt R, Bedi R (2004). Prevalence of self-assessed tooth discolouration ion the United Kingdom. J Dent.

[4] Alkhatib MN, Holt R, Bedi R (2005). Age and perception of dental appearance and tooth colour. Gerodontology.

[5] Xiao J, Zhou XD, Zhu WC, Zhang B, Li JY, Xu X (2007). The prevalence of tooth discolouration and the self-satisfaction with tooth colour in a Chinese urban population. J Oral Rehabil.

[6] Odioso LL, Gibb RD, Gerlach RW (2000). Impact of demographic, behavioural, and dental care utilization parameters on tooth colour and personal satisfaction. Compend Contin Educ Dent.

[7] Watts A, Addy M (2001). Tooth discolouration and staining: a review of the literature. Br Dent J.

[8] Joiner A (2006). Review of the extrinsic stain removal and enamel/dentine abrasion by a calcium carbonate and perlite containing whitening toothpaste. Int Dent J.

[9] Ten Bosch JJ, Coops JC (1995). Tooth colour and reflectance as related to light scattering and enamel hardness. J Dent Res.

[10] Nathoo S (1997). The chemistry and mechanisms of extrinsic and intrinsic discolouration. J Am Dent Assoc.

[11] Lendenmann U, Grogan J, Oppenheim FG (2000). Saliva and dental pellicle—A review. Adv Dent Res.

[12] Joiner A (2006). The bleaching of teeth: a review of the literature. J Dent.

[13] Griffiths CE, Bailey JR, Jarad FD, Youngson CC (2008). An investigation into most effective method of treating stained teeth: an *in vitro* study. J Dent.

[14] Sarrett DC (2002). Tooth whitening today. J Am Dent Assoc.

[15] Lynch CD, McConnell RJ (2003). The use of microabrasion to remove discoloured enamel: a clinical report. J Prosthet Dent.

[16] Walsh TF, Rawlinson A, Wildgoose D, Marlow I, Haywood J, Ward JM (2005). Clinical evaluation of the stain removal ability of a whitening dentifrice and stain controlling system. J Dent.

[17] Langford J, Pavey KD, Olliff CJ, Cragg PJ, Hanlon GW, Paul F, Rees GD (2002). Real-time monitoring of stain formation and removal on calcium hydroxyapatite surfaces using quartz crystal sensor technology. Analyst.

[18] Sulieman M, Addy M, Rees JS (2003). Development and evaluation of a method in vitro to study the effectiveness of tooth bleaching. J Dent.

[19] Stookey GK, Burkhard TA, Schemehorn BR (1982). *In vitro* removal of stain with dentifrices. J Dent Res.

[20] Addy M, Goodfield S (1991). The use of acrylic to compare the abrasivity and stain removal properties of toothpastes. Clin Mater.

[21] Lee BS, Huang SH, Chiang YC, Chien YS, Mou CY, Lin CP (2008). Development of in vitro tooth staining model and usage of catalysts to elevate the effectiveness of tooth bleaching. Dent Mater.

[22] Freccia WF, Peters DD (1982). A technique for staining extracted teeth: a research and teaching aid for bleaching. J Endod.

[23] Wetter NU, Barroso MC, Pelino JEP (2004). Dental bleaching efficacy with diode laser and LED irradiation: an in vitro study. Lasers Surg Med.

[24] Dawson PL, Walsh JE, Morrison T, Grigor J (1998). Dental stain prevention by abrasive toothpastes: a new in vitro test and its correlation with clinical observations. J Cosmet Sci.

[25] Parry J, Harrington E, Rees GD, McNab R, Smith AJ (2008). Control of brushing variables for the *in vitro* assessment of toothpaste abrasivity using s novel laboratory model. J Dent.

[26] Bollen CML, Lambrechts P, Quirynen M (1997). Comparison of surface roughness of oral hard materials to the threshold surface roughness for bacterial plaque retention: a review of the literature. Dent Mater.

[27] Shellis RP, Addy M, Rees GD (2005). *In vitro* studies on the effect of sodium tripolyphosphate on the interactions of stain and salivary protein with hydroxyapatite. J Dent.

[28] Hughes N, Maggio B, Sufi F, Mason S, Kleber CJ (2009). A comparative clinical study evaluating stain removal efficacy of a new sensitivity whitening dentifrice compared to commercially available whitening dentifrice. J Clin Dent.

[29] Porciani PF, Perra C, Grandini S (2010). Effect on dental occurrence by chewing gum containing sodium tripolyphosphate—a double-blind six-week trial. J Clin Dent.

[30] Alshara S, Lippert F, Eckert GJ, Hara AT (2013). Effectiveness and mode of action of whitening dentifrices on enamel extrinsic stains. Clin Oral Investig.

